# Knowledge and behavior of Nigerian dentists concerning the treatment of children with special needs

**DOI:** 10.1186/1472-6831-6-9

**Published:** 2006-06-19

**Authors:** Folakemi A Oredugba, Oluwatosin O Sanu

**Affiliations:** 1 Department of Child Dental Health, College of Medicine, University of Lagos, PMB 12003, Idi-Araba, Lagos, Nigeria

## Abstract

**Background:**

Children with special needs (CSN) are reported to receive less adequate dental care due to various behavioral problems and barriers created by dental professionals. This study was carried out to determine the knowledge and behaviour of Nigerian dentists concerning the treatment of CSN.

**Methods:**

Questionnaires consisting of open and closed ended questions requesting socio-demographic information, type of practice, undergraduate and postgraduate training, self-rated knowledge and behaviour concerning care of CSN, were hand delivered to 359 dentists in the 3 geographical zones of Nigeria over a period of 8 weeks. Responses were compared across age groups, gender, type of practice and training received.

**Result:**

Two hundred and eighty questionnaires were returned completed, constituting 79.9% response rate. Most of the respondents were aged 30 – 39 years (44.3%). There were more males (56.1%) and more recent graduates of 10 years and below (78.5%). Over 80% of respondents had treated children with disabilities, those with physical disabilities being most encountered. Only 19.3% of respondents rated their knowledge of management of CSN as adequate, with no significant difference across age groups and gender, but with a significantly higher number of older graduates reporting to have adequate knowledge (p < 0.05). Those who had undergraduate training in care of CSN were 69.5% compared with only 12.8% who had post graduate training. Only 11.8% rated their undergraduate training as adequate. Thirty seven percent of respondents rated the CSN they had treated as very challenging. A higher proportion of older graduates (of more than 10 years post graduation) and those who rated their undergraduate training as inadequate used sedation and general anaesthesia. Seventy one percent of respondents were willing to treat CSN, with no significant difference across age groups, gender and training, but with a significantly higher percentage among those who had rated their knowledge as adequate. Most of those who were unwilling to treat CSN felt their management was tedious and challenging.

**Conclusion:**

From this study, very few dentists reported to have adequate knowledge of management of CSN, irrespective of age, gender and place of practice. A significant number of those with more experience rated their knowledge as adequate. Although most dentists rated the children's behaviour as challenging, they indicated their willingness to treat them in their practices.

## Background

The provision of high quality dental care for children with special needs (CSN) presents a challenge for the dental profession [1]. CSN are those with a chronic physical, developmental, behavioral or emotional condition that substantially limits one or more major life activities, and who need dental or health services beyond what is generally required [2]. Some of such conditions are learning and developmental disorders, for example Down syndrome, cerebral palsy, epileptic or seizure disorders, vision and hearing impairment, HIV infection, cleft lip and palate, and other cranio-facial conditions. The chronicity of oral diseases such as dental caries and periodontal diseases complicates the primary disability [3].

Behavioral and co-ordination problems often exist within the dental environment, resulting in poor cooperation from this group of children [4]. The level of disability and patient behavior are obstacles to their receiving dental care [1]. In a study assessing the behavior and co-ordination of 2082 handicapped children aged 3–16 years, 53% were manageable in a normal dental surgery and 79% had the necessary coordination for routine dental care [4]. Of 744 children with special needs treated in another report, 90% were treated satisfactorily in the mobile clinic with cooperation in about 79%. Only 2% were totally uncooperative and had a general anaesthetic for ordinary examination and 8.5% received some treatment under general anaesthesia [5].

Their dental care could be seen as part of general dentists' professional responsibilities, and a vital course in the training of the paedodontist, though for the severely affected, a team approach to dental care is ideal [6]. Only a minority of such children are seen routinely in dental practice in Nigeria compared with the usual child population. Those who attend dental clinics only do so because of some symptoms. There are many issues to be examined concerning this trend: professional, parental and societal influences. In developed countries, it has been reported that many dental practitioners were reluctant to provide dental services for children with mental retardation, due to a combination of limited training, experience and interest as well as unrealistic financial reimbursement [7]. Training and experience are closely related to whether or not a child with disability is treated [8]. Parental attitudes and beliefs also determine whether a child receives dental care [9], so also the way the society views disability and supports families with such responsibilities [10].

This study was carried out to determine the knowledge and behavior of Nigerian dentists concerning the treatment of CSN so as to provide information necessary for curriculum review in our training institutions in Nigeria.

## Methods

### Respondents

Respondents were all Nigerian dentists practicing in the three geographical zones of the country – the North, South East and South West zones. All dentists that could be reached in their practices were included in the study.

### Materials

These were questionnaires containing 18 open- and close- ended questions which sought to provide information about the background variables of the dentist (age, gender, year of graduation), type of practice, (private, university/teaching or health center), whether the dentist received training in management of children with special needs at undergraduate or postgraduate levels and rating of such training. Other information required were self rated knowledge and behavior concerning such patients, methods employed in management, their rating of their management skills, willingness to treat such children or if referral was preferred and if parents of such children should be made to pay for oral health care services.

### Procedure

Ethical clearance for the study was obtained from the Research and Ethics Committee of the College of Medicine, University of Lagos. The survey was anonymous and the questionnaire was pre-tested on the first 10 dentists who received the questionnaire to check for clarity and ease of answering the questions. Others were delivered by hand by both authors to other dentists at their places of practice and collected over a period of 8 weeks.

### Statistical analyses

Data were entered into a computer and analyzed using Epi info version 6 [11]. Descriptive statistics and Chi square test of association were applied where appropriate with significance level set at p < 0.05.

## Result

Out of the 359 questionnaires given out, 280 were returned completed, a response rate of 79.9%. Respondents consisted of 56.1% males and 43.9% females. Majority (44.3%) was aged between 30 and 39 years, 36.4% were below 30 years while only 19.3% were above 40 years. More than half of the respondents (51.4%) were recent graduates of less than 5 years post graduation. One hundred and seventy-two dentists (61.4%) practiced in the teaching hospitals, 25.4% in state health centres and 13.2% in private establishments.

Gender and place of practice did not influence any of the parameters examined in the study. Majority of the respondents (83.6%) had treated CSN previously. Children with physical disabilities were most encountered by the dentists (39.3%) compared with other disabilities (p < 0.05).

Only 19.3% of the dentists rated their knowledge of management of CSN as adequate. One hundred and eighty-one (64.6%) of the respondents rated their knowledge as fairly adequate, with no significant difference among the three age groups and gender (Table 1). Thirty-five per cent and 44.5% of the dentists who graduated 16–20 years and > 20 years respectively rated their knowledge as adequate, compared with less than 20% of more recent graduates (Table 1). Among the respondents, 69.5% had undergraduate training in care of patients with special needs, with a significant proportion among recent graduates (10 years and below post graduation) (p = 0.014). A large proportion of this category of respondents also rated their undergraduate training as fairly adequate (p = 0.005) (Table 2). Only 12.8% of respondents had received post graduate training in the management of CSN and most of them were in the 6–15 years post graduation group.

**Table 1:** Distribution of Respondents' self-rated knowledge of management of CSN according to age and years post graduation

	Knowledge of management				
**Age (years)**	Don't know (%)	Adequate (%)	Fairly adequate (%)	Inadequate(%)	Total (%)

<30	0	16 (15.7)	73 (71.6)	13 (12.7)	102
30–39	1 (0.8)	21 (16.9)	79 (63.7)	23 (18.5)	124
>40	0	17 (31.5	29 (53.7)	8 (14.8)	54

Chi sq 9.47 p = 0.14					

**Years post graduation**					

<5	0	27 (18.7)	94 (65.3)	23 (16.0)	144
6–10	1 (1.3)	10 (13.2)	54 (71.0)	11 (14.5)	76
11–15	0	2 (9.1)	17 (77.3)	3 (13.6)	22
16–20	0	7 (35.0)	7 (35.0)	6 (30.0)	20
>20	0	8 (44.5)	9 (50.0)	1 (5.5)	18

Chisq = 22.1 p = 0.03*					

**Total**	**1 (0.4)**	**54 (19.3%)**	**181 (64.6)**	**44 (15.7)**	**280 (100.0)**

*Significant

**Table 2:** Distribution of respondents' years post-graduation according to undergraduate training, self-rating of undergraduate training and post graduate training.

Years post graduation						
Years	<5 (%)	6–10(%)	11–15(%)	16–20(%)	>20(%)	Total (%)

**Undergraduate training**						

Yes	112 (77.8)	51 (67.1)	12 (54.5)	10 (50.0)	10 (55.5)	195(69.5)
No	32 (22.2)	25 (32.9)	10 (45.5)	10 (50.0)	8 (44.5)	85(30.4)

Chi sq = 12.45 p = 0.01*						

**Self-rating of undergraduate training**						

Don't know	27 (18.8)	23 (30.3)	10 (45.5)	6 (30.0)	8 (44.4)	74 (26.4)
Adequate	20 (13.9)	7 (9.2)	1 (4.5)	4 (20.0)	1 (5.6)	33 (11.8)
Fairly adequate	80 (55.6)	39 (51.3)	10 (45.5)	2 (10.0)	7 (38.9)	138(49.3)
Inadequate	17 (11.8)	7 (9.2)	1 (4.5)	8 (40.0)	2 (11.1)	35 (12.3)

Chisq = 34.53 p = 0.00*						

**Postgraduate training**						

Yes	3 (2.1)	15 (19.7)	8 (36.4)	4 (20.0)	6 (33.3)	36 (12.8)
No	141 (97.9)	61 (80.3)	14 (63.6)	16 (80.0)	12 (66.7)	244(87.2)

Chi sq 36.63;p = 0.00*						

**Total**	**144 (51.5)**	**76 (27.1)**	**22 (7.9)**	**20 (7.1)**	**18 (6.4)**	**280 (100)**

*Significant

There was no significant difference among respondents' age groups and years post- graduation in their rating of management of CSN, but a significant number of those who rated their knowledge of management as inadequate felt their management was very challenging (p = 0.01) (Table 3). Respondents' rating of the CSN they had treated was also not influenced by age group or year of graduation. A significant number of those who rated their knowledge of management as fairly adequate described the children they had treated as uncooperative (Table 4). A higher proportion of older dentists, those who had graduated for more than ten years and those who claimed they had inadequate training in the management of CSN used sedation and GA for their patients (Table 5).

**Table 3:** Respondents' rating of management of CSN according to self rated knowledge of management.

Rating of management of CSN						
**Knowledge of management**	Very Challenging (%)	Challenging (%)	Challenging but interest-ing (%)	Burden-some (%)	Unintere-sting (%)	Total

Don't know	1 (0.4)	0	0	0	0	1
Adequate	20 (38.2)	14 (25.5)	19 (34.5)	1 (1.8)	0	54
Fairly adequate	64(35.4)	48 (26.5)	65 (35.9)	4 (2.2)	0	181
Inadequate	19(43.2)	7 (15.9)	12 (27.3)	3 (6.8)	3 (6.8)	44

Chi sq = 28.78 p = 0.01*						

**Total**	**104(37.2)**	**69(24.5)**	**96 (34.3)**	**8 (2.9)**	**3 (1.1)**	**280 (100)**

*Significant

**Table 4:** Respondents' rating of the CSN they had treated according to years post graduation and self rated knowledge of management

Rating of treated children				
**Years post-graduation**	Don't know (%)	Cooperative(%)	Uncooperative(%)	Total
<5	11 (7.6)	55 (38.2)	78 (54.2)	144
6–10	9 (11.8)	34 (44.7)	33 (43.4)	76
11–15	1 (4.5)	10 (45.5)	11 (50.0)	22
16–20	3 (15.0)	7 (35.0)	10 (50.0)	20
>20	0	7 (38.9)	11 (61.1)	18

Chisq = 19.49 p = 0.07				

**Knowledge of management**				

Don't know			1 (100.0)	1
Adequate	2 (3.6)	26 (47.3)	27 (49.1)	55
Fairly adequate	4 (2.2)	75 (41.4)	102 (56.4)	181
Inadequate	18 (41.9)	12 (27.9)	13 (30.2)	43

Chisq = 83.0 p = 0.0*				

**Total**	**24 (8.6)**	**113 (40.3)**	**143 (51.1)**	**280 (100)**

*Significant

**Table 5:** Behavior control methods used according to age group, years post graduation and self-rating of undergraduate training

Behaviour control method					
	No response (%)	Non-pharm(%)	Sedation(%)	Gen Anae(%)	Total

**Age group (years)**					

<30	6 (5.9)	79 (77.5)	16 (15.7)	1 (0.9)	102
30–39	14 (11.3)	83 (66.9)	25 (20.2)	2 (1.6)	124
>40	3 (5.6)	31 (57.4)	17 (31.5)	3 (5.5)	54

Chi sq = 12.7 p = 0.04*					

**Years post graduation**					

<5	11 (7.6)	103 (71.5)	28 (19.4)	2 (1.4)	144
6–10	8 (10.5)	54 (71.1)	13 (17.1)	1 (1.3)	76
11–15	0	15 (68.2)	7 (31.8)	0	22
16–20	4 (20.0)	10 (50.0)	6 (30.0)	0	20
>20	0	11 (61.1)	4 (22.2)	3 (16.7)	18

Chi sq = 30.6 p = 0.00*					

**Rating of undergraduate training**					

Don't know	6 (8.1)	52 (70.3)	14 (18.8)	2 (2.7)	74
Adequate	0	26 (78.8)	7 (21.2)	0	33
Fairly adequate	7 (5.1)	99 (71.7)	28 (20.3)	4 (2.9)	138
Inadequate	10 (28.6)	16 (45.7)	9 (25.7)	0	35

Chi sq = 27.8 p = 0.00*					

**Total**	**23 (8.2)**	**193 (69.0)**	**58 (20.7)**	**6 (2.1)**	**280**

*Significant

Among all the respondents in this study, 76.8% were willing to take up the challenges of treatment of the children, irrespective of age and type of undergraduate training. Twenty one percent females and 24.8% males would refer CSN to other practitioners for treatment. There was no significant difference in the proportion of those willing to treat CSN and those not willing across age groups and whether or not respondents had the undergraduate training. A higher proportion of those who rated their knowledge as adequate and fairly adequate were willing to treat the children while a higher proportion who rated their knowledge as inadequate were not willing to treat (p < 0.05) (Table 6).

**Table 6:** Distribution of respondents willing to treat CSN according to self-rated knowledge of management.

Willingness to treat			
**Knowledge of management**	Yes	No	Total

Don't know	1 (100.0)	0	1
Adequate	45 (83.3)	9 (16.7)	54
Fairly adequate	135 (74.6)	46 (25.4)	181
Inadequate	20 (45.5)	24 (54.5)	44

Chi sq = 17.19 p = 0.00*			

**Total**	**201 (71.8)**	**79 (28.2)**	**280 (100.0)**

*Significant

**Figure 1:**
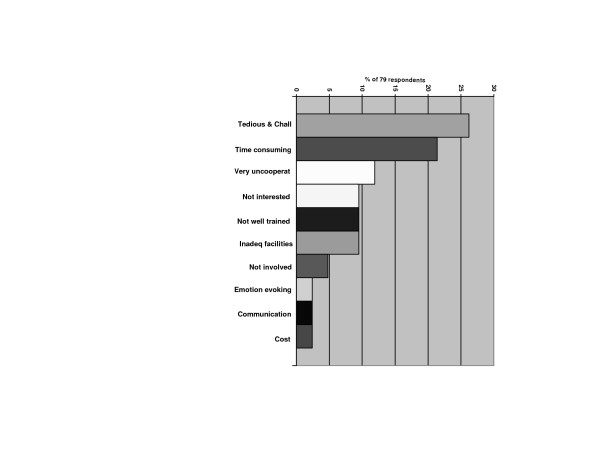
Reasons given by some respondents for not willing to treat CSN.

Overall, 54.6% would not want parents of the children to pay for dental services, with a significant proportion with such opinion among the older dentists (p < 0.05). Most of the dentists not willing to treat CSN claimed their management was tedious and challenging (26.2%), time consuming (21.4%) and that the children were uncooperative (11.9%). Others (9.5%) were 'not interested', 'not well trained' and 'did not have adequate facilities' to treat them. Remuneration for treatment (cost) was low on the list of reasons (Figure 1).

## Discussion

More than half of respondents in this study reported that they had fairly adequate knowledge of the management of children with special needs (CSN). A higher proportion in the older age group had adequate knowledge of management of CSN, though these differences were not significant across age groups and gender. Experience in this field is apparently gained with increasing years of practice. This trend is obvious when we considered the level of knowledge according to the number of years post graduation of the respondents. A significant proportion of those who had graduated over sixteen years had adequate knowledge of management of CSN. Provision of oral health care to children and adolescents with special health care needs requires specialized knowledge, increased awareness and attention, and accommodation [12].

A significant proportion of recent dental graduates responded that they had undergraduate training in the management of CSN which they also rated fairly adequate. The dental schools are responding to the need for training dentists in this special field. For example in the Dental School of the University of Lagos, more than 12 hours are currently devoted to classroom teaching and clinical exposure in care of CSN, up from only 6 hours previously. Arrangements are also being made to include community contact of final year dental students with institutions for individuals with special needs.

It was also observed from this study, that a third to a fifth of the older graduates had received post graduate training in the care of CSN. An extended course of post graduate study is required to train dentists to manage patients with severe disabilities [3]. The post-graduate course in paediatric dentistry also involves training in the care of children with disabilities.

Most of the respondents rated the management of the children as 'challenging' and 'challenging but interesting' regardless of their age group and number of years post graduation. Adequate exposure to such children and their problems during training will reduce anxiety and dispel the fears experienced by practicing dentists. Over 50% of dentists surveyed also saw the children as uncooperative, with no significant difference across age groups and years of graduation. This is a general belief, but responses also depend on the type of disability encountered. Most physical disabilities may prevent children from carrying out their routine oral hygiene and so may reduce compliance with dental instructions. They may also be prevented from keeping hospital appointments. Majority of dentists surveyed used the non pharmacological method of behavior control more frequently than sedation and general anaesthesia. Older dentists also used sedation more than the younger ones. The use of sedation and general anaesthesia, apart from the extra cost, also carries some risks which may not be easily managed by untrained hands. Facilities must be available to manage the patient because of uncertain outcome of morbidity and mortality [13,14]. More dentists who claimed they did not have adequate training in care of CSN used sedation more than others with adequate or fairly adequate training. Since the former group may not be trained to use the non pharmacological method for CSN, sedation, which requires little dentist-patient communication, may be a more comfortable technique for that group. Many dentists in other countries have been reported to have low confidence in their ability to manage patients with special needs [15]. This is a direct result of little or no training in special care dentistry in many oral health undergraduate programs [16].

The learning of appropriate interpersonal communication skills has been reported to be a crucial part of a health care professional's education [17]. From the result of this survey, very few Nigerian dentists used general anaesthesia for CSN. This shows that those who required complex treatment and whose behaviour is extremely difficult to manage in the regular clinics may go without adequate treatment or managed with sedation. Special care in dentistry also includes managing or accommodating the behaviour of a resistant patient and making modifications to routine treatment procedures [18]. This eliminates the challenging behaviour that is known with such patients and reduces the time spent in treatment, which tops the list of reasons given for the dentists' unwillingness to treat. Special training is required in this field to help interested dentists to cope with difficult behaviour [3]. However, most Nigerian dentists in this study were willing to face the challenges of management, irrespective of their basic training and type of practice. It may be assumed from this study that, given adequate facilities, training support and good remuneration, they will be able to give better care to such children.

Most of the respondents were willing to treat CSN, irrespective of their age groups, gender, undergraduate and postgraduate training, though with slightly fewer older dentists. This contrasts with other reports that older dentists were more likely to provide care for CSN [19].

A significant proportion of those who had adequate and fairly adequate knowledge of management of CSN were also willing to treat CSN. Those not willing to treat may lack information and previous exposure to this group of patients. Professional barriers to treating such patients have been reported to include inadequate undergraduate education, inadequate staff training, failure to recognize diversity and see oral health in the context of illness and disability [20]. An undergraduate course will provide the opportunity for students to have contact with such patients and to confront their fears and anxieties about treating them in future [21]. In this study, dentists' practice settings did not influence their response to willingness to treat CSN. Previous studies have reported that dentists practicing in dental schools and teaching institutions are more willing to treat such children. This may be because of the availability of better equipment and support staff. Most dental establishments in Nigeria have the basic equipment to handle their management, so the dentists' unwillingness to treat the children is a barrier relating to the dentists' disposition. This may depend on the amount of training received by the health professional, the working environment and the role adopted by the health care worker [22]. A 2003 survey conducted by the World Dental Federation (FDI) revealed that employers of dental school graduates felt they were less competent to provide services to individuals with special health care needs [23]. If training is not adequate, as evidenced by findings in this study, the practitioner might not be well disposed to handling this group of children. However it was noted from this study that majority of the respondents, irrespective of gender, age group and years post graduation, had treated CSN at one time or the other in their practices. Most dentists also felt that they should not be compelled to pay for dental services. In their opinion, the families of such children are already financially burdened with other medical and social services so the cost of expensive routine dental care may be waived. Fortunately, the cost of treatment was not a major problem for the respondents in this survey. High on the list of barriers to providing care for this group of children were the tedious and challenging process in the clinic, time consumption and the uncooperative behaviour of the children. Improved training will help the dentist to better manage patient behaviour and utilize time effectively. Provision of adequate facilities and a revised curriculum, which should also incorporate community outreach for students will stimulate their interest and therefore improve care for CSN in Nigeria.

## Conclusion

It may be concluded from this study that very few dentists reported to have adequate knowledge of management of CSN, irrespective of age, gender and place of practice. A significant number of those with more experience rated their knowledge as adequate. Although most dentists rated the children's behaviour as challenging, they indicated their willingness to treat them in their practices.

## Competing interests

The author(s) declare that they have no competing interests.

## Authors' contributions

FAO designed the questionnaire, participated in the coordination, performed the statistical analysis and drafted the manuscript. OOS conceived of the study and participated in the design and coordination. Both authors read and approved the final manuscript.

## Pre-publication history

The pre-publication history for this paper can be accessed here:


